# A rare case of heterotopic pancreatitis and intestinal malrotation in a COVID-19 positive patient. COVID-19, causative or coincidence?

**DOI:** 10.1016/j.ijscr.2021.105917

**Published:** 2021-04-27

**Authors:** Ivan Tomasi, Luca Scott, Jack Cullen, Francesco di Maggio, Husam Ebied, Sarah Wheatstone

**Affiliations:** Department of Emergency General Surgery, Guy's and St Thomas' Hospital London, Westminster Bridge Road, London SE1 7EH, United Kingdom of Great Britain and Northern Ireland

**Keywords:** HP, heterotopic pancreas, SARS-CoV-2, severe acute respiratory syndrome corona virus 2, COVID-19, coronavirus disease 2019, ED, Emergency department, DJ, duodeno-jejunal, CT, computed tomography, MRI, magnetic resonance imaging, ACE2, angiotensin-converting enzyme 2, PPE, personal protective equipment, AGPs, aerosol generating procedures, COVID-19, Case report, Heterotopic pancreatitis

## Abstract

**Introduction and importance:**

Heterotopic pancreas (HP) is defined as the presence of pancreatic tissue without anatomical and vascular continuity with the main body of the pancreas. HP typically remains asymptomatic, however complications such as acute pancreatitis can arise. Gastrointestinal involvement with coronavirus disease 2019 (COVID-19) is not uncommon and there are reported cases of associated pancreatitis.

**Case presentation:**

A 31-year-old male presented to the Emergency department (ED) with a 3-day history of right iliac fossa pain. The patient was found to have COVID-19 and a planned laparoscopic appendectomy was later converted to a midline laparotomy when a mass close to the duodeno-jejunal (DJ) flexure was identified. Following a hand-sewn anastomosis the patient made a good post-operative recovery. Histology illustrated the presence of heterotopic pancreatic tissue within the small bowel with underlying fat necrosis typical of acute pancreatitis. Follow-up radiology supported the intraoperative finding of intestinal malrotation.

**Clinical discussion:**

Rarely the combined presence of intestinal malrotation and HP in patients has been noted. Heterotopic pancreatitis can present in a multitude of ways and it is a difficult diagnosis to make pre-operatively. Emerging literature documents the potential presentation of COVID-19 with acute pancreatitis. The expression of angiotensin-converting enzyme 2 (ACE2) receptors on the pancreas is believed to play a role.

**Conclusion:**

This is the first documented case of heterotopic pancreatitis with intestinal malrotation in a COVID-19 positive patient. We hypothesise that the COVID-19 infection contributed to the heterotopic pancreatitis.

## Highlights

• Heterotopic pancreas can be present in up to 13.7% of patients.
• Heterotopic pancreatitis is a rare complication.
• Pre-operative diagnosis of heterotopic pancreatitis is difficult.
• COVID-19 can induce pancreatic injury and potentially acute pancreatitis.
• COVID-19 may have contributed to the heterotopic pancreatitis in our patient.

## 1 Introduction

Heterotopic pancreas (HP) or ectopic pancreas is defined as the presence of pancreatic tissue isolated anatomically from the main body of the pancreas, with independent ducts and vascular supply. This rare congenital condition is well reported in the literature as far back as 1727, with first histological confirmation in 1859 [1]. HP is documented in between 0.5 and 13.7% of autopsy studies and identified in 0.5% of laparotomies [2,3].

HP typically remains asymptomatic and is often found incidentally. However, complications such as inflammation, bleeding, obstruction and malignant transformation can all arise [1]. Acute heterotopic pancreatitis is rare, normally presenting with abdominal pain and can cause an increase in serum pancreatic enzymes [4,5].

The novel severe acute respiratory syndrome corona virus 2 (SARS-CoV-2) has resulted in the coronavirus disease 2019 (COVID-19) pandemic. COVID-19 has primarily been discussed as disease affecting the respiratory system, however gastrointestinal involvement and more specifically pancreatic injury can also occur [6,7].

We present a rare case of heterotopic pancreatitis in a COVID-19 positive patient reported in line with the SCARE criteria [8]. To the best of our knowledge, this is a unique case with no similar reports in the medical literature.

## 2 Presentation of case

A 31-year-old male patient, originally from Angola, presented to the Emergency department (ED) with a 3-day history of right iliac fossa pain associated with bilious vomiting. He had been opening his bowels normally with formed stools. This patient had no significant past medical history and was not taking any regular medications. He was a non-smoker, drinking around 15 units of alcohol a week.

On presentation to the ED he was haemodynamically stable and apyrexial. He reported a pain of 4 on a severity scale of 1 to 10. Physical examination revealed rebound tenderness in the right iliac fossa with no palpable masses, and was otherwise unremarkable.

A routine SARS-CoV-2 test (polymerase chain reaction - PCR) performed in the ED showed positivity. Urinalysis and a plain film chest radiograph illustrated no abnormalities. His blood tested read; WBC 11.0 (4.0–11.0 × 10^9) Neutrophils 7.7 (1.5–7.0 × 10^9), CRP 55 (0–4 mg/L), Amy 39 (0–99 IU/L) Lactate 0.83 (0.20–1.80 mmol/L), INR 1.2 (0.8–1.2 ratio), Ferritin 659 (30–400 μg/L), Urea 2.8 (1.7–8.3 mmol/L), Creatinine 65 (59–104 μmol/L), eGFR 124 (70–130 mL/min).

The patient was admitted under the general surgical team with suspected appendicitis and was given cefuroxime and metronidazole. The diagnosis of acute appendicitis was made clinically. To reduce radiation exposure in young patients, in our centre, pre-operative computed tomography (CT) imaging is reserved for cases of diagnostic uncertainty. He was consented for a laparoscopic appendectomy and taken to theatre by a consultant surgeon.

The patient remained in a side room on the ward and appropriate personal protective equipment (PPE) was worn during all interactions with the patient as per local guidelines. Similarly, in line with guidance for aerosol generating procedures (AGPs) the patient and the anaesthetist remained in the anaesthetic room for 20 min following tracheal intubation prior to the operation.

**Fig. 1:**
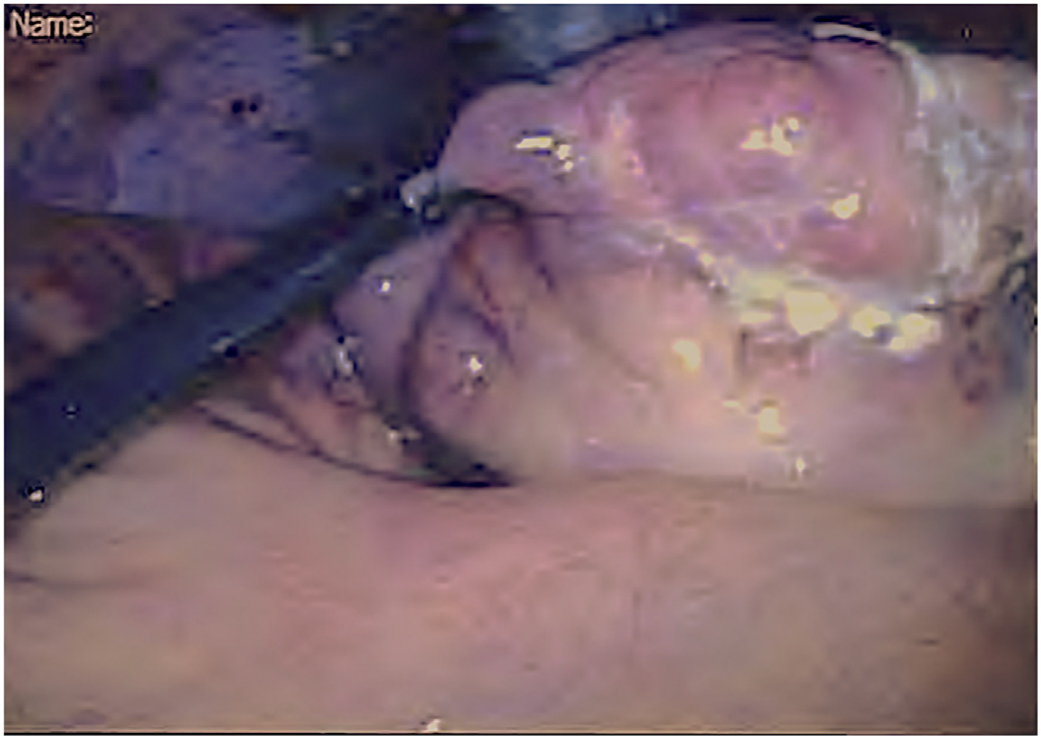
Intra-operative laparoscopic picture, illustrating the proximal small bowel mass superiorly and malrotated mesentery inferiorly.

Intra-operatively a small intestinal loop mass at 20 cm distal to the duodeno-jejunal (DJ) flexure with free purulent fluid was identified (Fig. 1). The procedure was converted to a midline laparotomy for a small bowel resection, during which an intestinal malrotation was noted. The aetiology of the mass was unclear; therefore, it was resected en-bloc with wide resection margins including associated mesentery. Hand-sewn end to end anastomosis 20 cm distal to the DJ flexure was made. The appendix appeared macroscopically inflamed and was also resected.

Frozen sections were not sent during the operation. In our institution, these are predominately available for elective surgery and in this case we do not believe they would have influenced management as the inflamed bowel required resection either way.

Specimens (small bowel 94 × 40 × 30 mm, adhesions 95 × 10 mm, appendix and mesoappendix 65 × 15 × 6 mm) were sent for histological analysis.

The patient made a good post-operative recovery without early complications. He was discharged on post-operative day three with well-controlled pain, tolerating oral intake and opening his bowels. He was advised to self-isolate for a further 10 days (14 days from the positive COVID-19 test) following discharge to help mitigate the risk of community COVID-19 transmission.

**Fig. 2:**
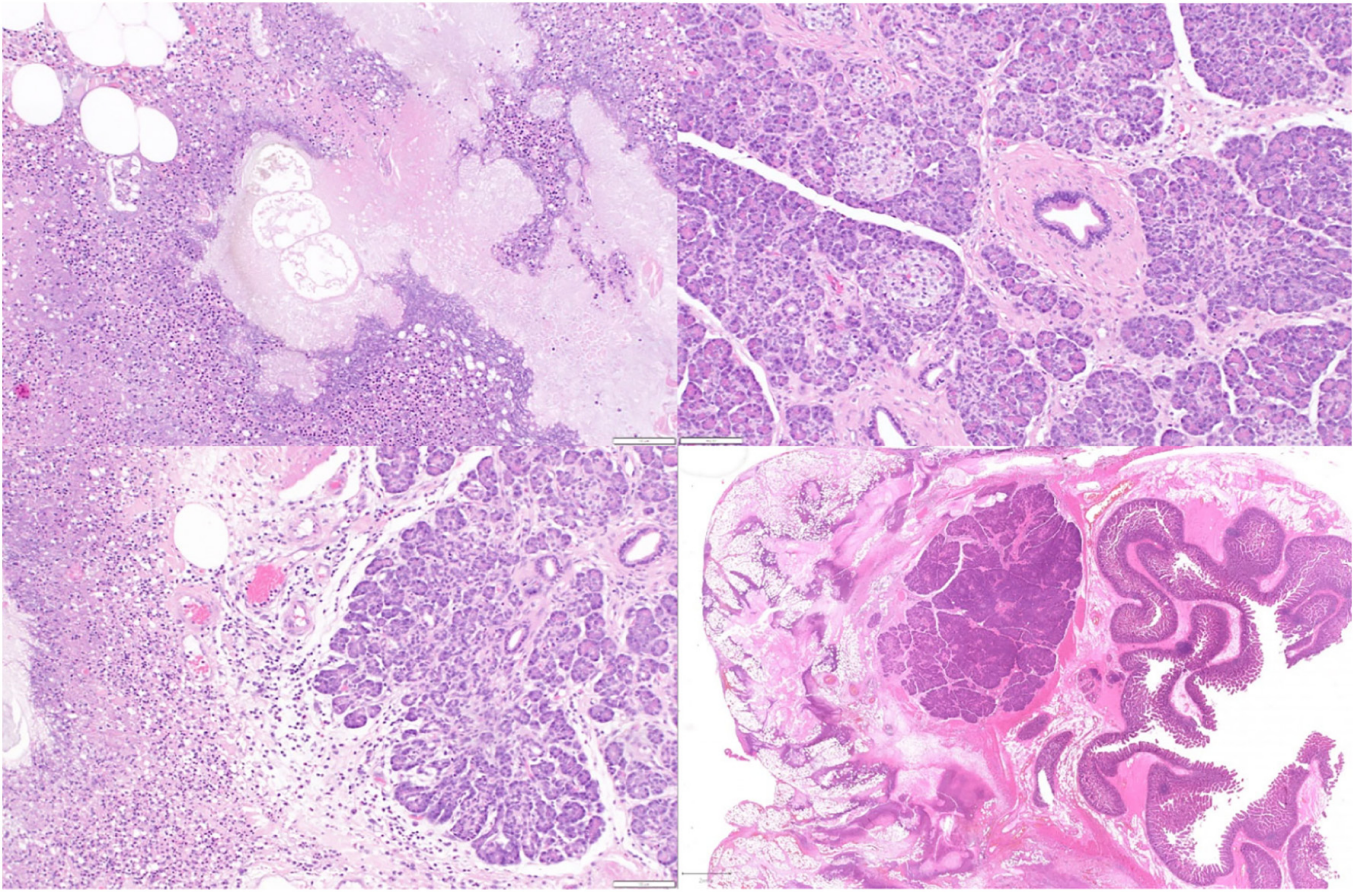
Representative images of Hematoxylin and Eosin (H&E) stained inflamed heterotopic pancreatic tissue and necrosis of underlying fat of small intestine sections.

Pathology reported the presence of heterotopic pancreatic tissue within the muscularis propria and submucosa of the small bowel. Within the limited focal inflammation of the ectopic pancreatic tissue, extensive necrosis and fibrinous serositis of the underlying fat typical of acute pancreatitis was noted (Fig. 2). Background small bowel and appendix were reported within normal histological limits.

**Fig. 3:**
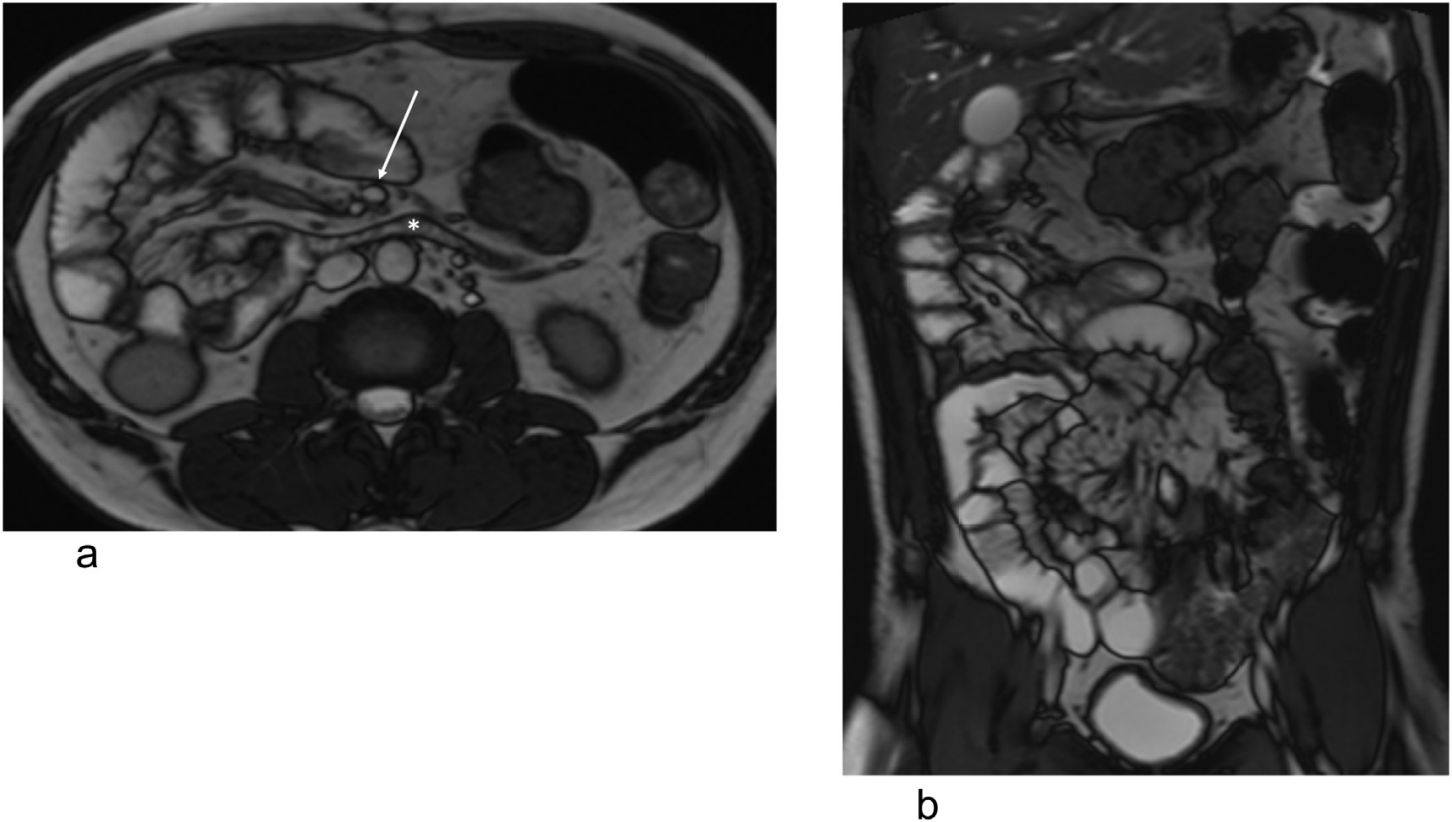
a- MRI, axial ‘True FISP’ image showing congenital small bowel malrotation with reversed orientation of the superior mesenteric vessels (arrow). Although the 3rd part of the duodenum (*) is normally sited, the DJ flexure and small bowel loops lie to the right of the midline with the colon seen on the left. b- MRI, coronal ‘True FISP’ image showing congenital small bowel malrotation with small bowel loops in the right side of the abdomen and colon on the left.

Follow-up was carried out remotely in response to the COVID-19 pandemic. COVID-19 tests were negative at 30 and 50 days post operation. Two months after the operation the patient was re-admitted with abdominal pain. A CT abdomen and magnetic resonance imaging (MRI) of the small bowel ruled out any further inflammatory processes of the abdominal viscera. The radiology supported the intraoperative findings of an intestinal malrotation (Fig. 3a and b). The patient was later discharged with a diagnosis of ‘adhesion pain syndrome’.

## 3 Discussion

While there are several proposed theories for the pathogenesis of Heterotopic pancreas (HP), the most commonly held belief is that pancreatic tissue from the ventral and dorsal buds is deposited at ectopic sites during the embryonic rotation of the foregut [1,3]. Interestingly, our patient was found to have a second abnormal embryonic variant – an intestinal malrotation. The incidence of midgut malrotation is 0.2% and is a deviation from the normal 270-degree counter-clockwise embryological rotation [9]. HP can coexist with other embryological abnormalities such as oesophageal atresia, Meckel diverticulum and intestinal malrotation [10].

While 70–90% of HP localize in the upper gastrointestinal tract (stomach 25–47%, duodenum 11.7–36.3% and proximal jejunum 15–35%), remote sites such as teratomas, fallopian tubes and the mediastinum have also been reported [1,2,11]. HP is predominately found in the submucosal layer and classified into three groups based on the presence in the ectopic tissue of ducts, acini and endocrine islets [1,2].

HP often remains asymptomatic, however as pancreatic tissue, it can undergo inflammation, bleeding, obstruction and malignant transformation [1]. In our case it resulted in acute ectopic pancreatitis within the upper-gastrointestinal tract, previously reported in several cases [1,2,4,5,12,13].

It is difficult in these cases to make an accurate pre-operative diagnosis. Our patient was believed to have an acute appendicitis; Rubesin et al. discuss a case of presumed jejunal diverticulitis and Mickuniene et al. document another case of small bowel HP characterized by bleeding and episodic abdominal pain. Symptomatic HP can indeed have variable presentations and should be considered a rare differential in cases of abdominal pain associated with diagnostic uncertainty [2,4]. Surgical management was required in the majority of the reviewed cases.

Whilst heterotopic pancreatitis is rare, the presence and potentially contributing role of COVID-19 makes our case unique. The novel SARS-CoV-2 RNA virus is responsible for the infectious disease COVID-19, which has resulted in a global pandemic [14]. SARS-CoV-2 penetrates host cells through binding to the angiotensin-converting enzyme 2 (ACE2) receptor on their surface [14].

Pancreatic involvement in COVID-19 has been reported, varying from an asymptomatic rise in pancreatic enzymes to acute pancreatitis [6]. Several reports have documented COVID-19 to occur with pancreatitis in the absence of other classical aetiological factors such as alcohol and gallstones [6,7]. Pancreatic islet cells express ACE2 receptors to which SARS-CoV-2 can bind, potentially resulting in direct cytopathic effect and pancreatic injury. Alternatively, indirect immune mediated damage may occur through cytokine storm and a systemic inflammatory response [6,14]. Inamdar et al. found on retrospective analysis a much higher incidence of idiopathic pancreatitis in patients with COVID-19 than in the normal population, suggesting a role for SARS-CoV-2 [15].

Our patient was known to drink 15 units of alcohol a week for several years. While acute alcohol intake is a common precipitant for pancreatitis, considering the aforementioned pathophysiological mechanism and the several reported cases of acute pancreatitis in COVID-19 patients, along with the extensive narrative of viral causes of pancreatitis, it seems reasonable to believe that COVID-19 has contributed to the pathology in our case [7]. Furthermore, the patient had no prior sequelae from his alcohol consumption before contracting COVID-19.

Discussion with local gastrointestinal pathologists with COVID-19 post-mortem histopathological experience proved interesting. The fat necrosis surrounding the ectopic pancreas was in-keeping with acute pancreatitis. However, there were no specific histological features normally associated with COVID-19, such as acute infarctions of the villous tips of the small bowel. An important limitation to this analysis is that COVID-19 stains currently work reliably only in the lungs, hence COVID-19 could still have contributed to the heterotopic pancreatitis despite the above histological findings.

## 4 Conclusion

We have reported the first case of heterotopic pancreatitis with intestinal malrotation in a COVID-19 positive patient. Pancreatitis of ectopic tissue is a rare phenomenon previously documented. In light of the emerging literature associating COVID-19 and pancreatitis, we hypothesise that the simultaneous presence of heterotopic pancreatitis and COVID-19 infection in our patient may not be a coincidental finding.

## Sources of funding

This research did not receive any specific grant from funding agencies in the public, commercial, or not-for-profit sectors.

## Ethical approval

N/A.

## Consent

Written informed consent was obtained from the patient for publication of this case report and accompanying images. A copy of the written consent is available for review by the Editor-in-Chief of this journal on request.

## Author contribution

Ivan Tomasi –Designed the study, collected and interpreted data and wrote the paper.

Luca Scott- Interpreted data and wrote the paper.

Jack Cullen- Collected data and reviewed the paper.

Francesco DiMaggio- Designed the study, interpreted data and wrote the paper.

Husam Ebied- Interpreted data.

Sarah Wheatstone -Designed the study and interpreted data.

## Research registration (for case reports detailing a new surgical technique or new equipment/technology)

N/A.

## Guarantor

Mr. Ivan Tomasi.

## Provenance and peer review

Not commissioned, externally peer reviewed.

## Declaration of competing interest

None.
